# Semi-quantitative determination of ash element content for freeze-dried, defatted, sulfated and pyrolysed biomass of *Scenedesmus* sp.

**DOI:** 10.1186/s13068-020-01699-8

**Published:** 2020-04-01

**Authors:** Rafael Augusto Sotana de Souza, Flávia Marisa Prado Saldanha-Corrêa, Antonio Garrido Gallego, Ana Maria Pereira Neto

**Affiliations:** 1 Centro de Engenharia, Modelagem e Ciência Sociais Aplicadas, Universidade Federal do ABC, Avenida dos Estados, 5001, Bairro Bangu, Santo André, SP 09210-580 Brazil; 2 Instituto Oceanográfico, Universidade de São Paulo, Praça do Oceanográfico, 191, Butantã, São Paulo, SP 05508-120 Brazil

**Keywords:** Microalgae, Lipid determination, Thermochemical conversion, Ash composition, Oxide formation

## Abstract

**Background:**

Energy demand by mankind has become one of the most important aspects of our society. A promising technology that seeks to provide part of the energy demand and to obtain high-value products is the thermochemical conversion of microalgae biomass. Inorganic species presented in microalgae biomass may act as catalysts for thermochemical reactions and are responsible for notorious ash-related issues during thermochemical decomposition.

**Results:**

In this study, the freeze-dried biomass of *Scenedesmus* sp. was used to evaluate the lipid extraction methodology regarding a sonication bath as pretreatment technique for cell disruption followed by vortex mixing and *n*-hexane as solvent. It is also presented the lipid and amino acid profiles for *Scenedesmus* sp. The freeze-dried biomass was pyrolysed through a TGA (thermogravimetric analysis), with heating rates of 20 °C/min, from 100 to 650 °C. The ash and sulfated ash contents were accurately determined by combustion of biomass in a muffle furnace. The element component of ashes of the freeze-dried, defatted, pyrolysed and sulfated biomasses was determined by means of scanning electron microscope (SEM) fitted with energy dispersive spectroscopy (EDS). The lipid content obtained for *Scenedesmus* sp. dry biomass was 16.72% (± 0.03). The content of the sulfated ash obtained was 17.81 ± 0.15%. The SEM–EDS technique identified different mineral compounds in ashes, allowing to quantify Mg, P, S, K, Ca, Fe, Co and Br, as well as oxides.

**Conclusion:**

The results suggest a possible strategy to evaluate in a semi-quantitative manner the ash composition of freeze-dryed, defatted, sulfated and pyrolysed biomass of *Scenedesmus* sp. and its feasibility in using *Scenedesmus* sp. biomass in different thermochemical conversion strategies to achieve processes with positive energy ratio, representing potential use both environmental and energetically.

## Background

Currently, most of the energy consumed in daily activities comes from non-renewable sources of energy, obtained through fossil fuels (coal, natural gas and oil). According to the International Energy Agency (IEA), the total primary energy supply between 1971 and 2017 increased approximately 2.5 times, starting from the value of 5523 million tonnes of oil equivalent (Mtoe) in 1971 to 14,034 Mtoe in 2017 [1]. Considering this current scenario, the 2030 *Agenda for Sustainable Development* [2] and the Brazilian Energy Plan [3], related to climate change and energy security issues, an alternative proposed for the reduction in the use of fossil fuels by the transport sector is the use of biofuels, already implemented in Brazilian energy matrix.

Microalgae appear as a renewable source of biofuels that could meet some of the global demand for energy. Microalgae can accumulate large deposits of oil in their cells, and the potential of some microalgae species as a source of renewable energy has received considerable interest around the world. In addition to that, the use of raw materials which not demand the use of agricultural areas is necessary to minimize competition for food production [4].

Microalgae are single-celled photosynthetic organisms that have high growth rates, about 100 times faster than terrestrial plants, doubling their biomass in a daily scale. For comparative purpose, they may require much less land area, up to 49 or 132 times less, when compared to rapeseed or soybean crops, used as biodiesel feedstock [5]. In addition, microalgae account for 40% of global carbon sequestration and they have the ability to thrive in animal waste, domestic wastewater, some industrial effluent, can be used in aquaculture, animal feed, and in obtainment of high-value bioproducts such as triglycerides, antioxidants, pigments, β-carotene, polysaccharides, fatty acids, including omega-3 and omega-6, vitamins, and being used in different sectors (biofuels, functional foods, pharmaceuticals, nutraceuticals, cosmetics) [5, 6].

Oil extraction from microalgae stands as a huge challenge for bioproducts’ and biofuels’ production, mainly due to high costs and energy requirement. Microalgae processing presents several bottlenecks due to facts like the low density of the microalgae that store lipids and that they are found in suspension which makes their separation difficult and expensive for large-scale production. The use of solvents is an effective method for total biomass separation [7].

Rizzo et al. [8] have already carried out a preliminary investigation in the characterization of the pyrolytic oil of the microalgae *Chlorella* and *Nannochloropsis* sp. This oil presented higher energy value, especially when compared to lignocellulosic biomasses (28.4 vs 17.2 MJ/kg), mainly due to the lower oxygen content in the composition, the carbon/hydrogen ratio similar to the diesel, low density and minimally basic pH. Raheem et al. [9] also conducted studies indicating that pyrolysed microalgae bio-oils are more stable and less oxygenated than bio-oils from lignocellulosic biomass.

He et al. [10] proposed that inorganic species inherently presented in defatted microalgae may act as catalysts for thermochemical reactions, being responsible for notorious ash-related issues. Obtaining data from these ashes’ elemental composition is essential for choosing the best technologies for microalgae processing, once the transformation of these elements during thermochemical conversions is strongly dependent on their speciation. Also, the knowledge of ashes’ elemental composition has the importance in developing and improving equipments such as boilers, gasifiers, etc. For example, in energy conversion plants, chlorine is the main element that promotes corrosion at high temperature and, consequently, low efficiency, since it is responsible for the formation of hydrochloric acid, which is responsible for air pollution and corrosion of equipment [11].

In this study, the lipid and ash contents and ash elemental composition of the freshwater microalgae *Scenedesmus* sp. is presented. The methodology of energy dispersive spectroscopy (for the freeze-dryed, defatted, sulfated and pyrolysed biomass) is proposed as a possible strategy to evaluate in a semi-quantitative manner the ash composition of microalgae biomass. The data for lipid profile and amino acid profile were supported by *Algae Biotecnologia*^®^.

## Results and discussion

Algal biomass has been reported as a feedstock with a high potential for biofuel production and higher productivity than terrestrial crops [5, 12].

The lipid content obtained for *Scenedesmus* sp. dry biomass was 16.72% (± 0.03). This result is similar to the one obtained by Thanh et al. [13], who cultivated *Scenedesmus quadricauda* with different air injection rates (among 5 to 65 L/min) and obtained a lipid content of 16% for the majority of these injection rates. Zhang et al. [14] obtained 13.8% (± 0.4) with an enzyme-assisted lipid extraction with cellulase, xylanase and pectinase for *Scenedesmus* sp. The lipid content reported by Rai and Gupta [15] for *Scenedesmus abundans* was 48% for autotrophic cultures in photobioreactors in the absence of nitrogen in the culture medium, suggesting that a higher lipid content can be obtained with this type of stress condition (depletion of nitrogen). For *Scenedesmus* sp., the data of the fatty acid profile are presented in Table 1.

**Table 1:** Fatty acid composition of *Scenedesmus* sp.

Fatty acid	Content (%)
C14:0	0.34
C15:0	0.13
C16:0	18.45
C16:1	0.61
C17:0	0.64
C17:1	2.65
C18:0	1.73
C18:1 Cis/Trans	6.35
C18:2 Cis/Trans	13.46
C18:3 n3	0.35
C18:3 n6	52.98
C20:0	0.1
C20:1(9)	0.12
C20:2 (11,14)	0.21
C20:3 (11,14,17)	0.94
C22:0	0.55
C24:0	0.4
Saturated	22.34
Unsaturated	77.67
Monounsaturated	9.73
Polyunsaturated	67.93

Microalgae have a greater diversification of fatty acids in their composition when compared to plants. In edible oleaginous plants, the main fatty acids found are lauric acid (C12:0), myristic acid (C14:0), palmitic acid (C16:0), stearic acid (C18:0), oleic acid (C18:1) and linolenic acid (C18:3) [16]. According to the results (Table 1), the fatty acids obtained for *Scenedesmus* sp. were on average composition of: 22.34% saturated and 77.66% unsaturated acids. For the unsaturated, 9.73% are monounsaturated and 67.93%, polyunsaturated. These values denote a high concentration of unsaturated fatty acids, negatively impacting the use of these oils for the production of biodiesel, as these compounds imply a lower oxidative stability to biodiesel, being necessary the addition of antioxidants. However, the use of *Scenedesmus* sp. should be evaluated, because a higher number of unsaturations imply a lower viscosity, providing adequate flow properties to the fuel, especially for countries with cold weather. Therefore, the amounts of saturated and unsaturated fatty acids influence the quality of the biodiesel, and can determine its commercialization potential [17].

According to Sawangkeaw and Ngamprasertsith [16], biodiesel derived from microalgae oil has low viscosity and oxidative stability compared to biodiesel produced with palm oil, for example. According to Schenk et al. [18], biodiesel quality is related to the palmitoleic (C16:1), oleic (C18:1) and myristic (C14:0) fatty acids, which give biodiesel a low oxidative potential, in a ratio of 5:4:1 respectively, maintaining satisfactory levels of fluidity and number of cetanes.

However, in relation to other important properties (density, flash point and calorific value), biodiesel obtained from the algae biomass has similar or superior fuel properties to diesel, as reported by Xu et al. [19], Suganya et al. [20] and Bagul et al. [21].

Regarding the fatty acids present in *Scenedesmus* sp., the most important are gamma linolenic acid (GLA) with 52.98% content (also present in *Spirulina platensis* [22]); linoleic acid (LA) with 13.46%. GLA is a precursor of prostaglandin E1, which has biological activity to reduce inflammatory processes and blood pressure. This fatty acid is also used to treat rheumatoid arthritis, eczema and premenstrual tension [22]. LA is the precursor of long chain polyunsaturated fatty acids in humans [≥ C20, for example, eicosapentaenoic acid (EPA, C20:5); docosahexaenoic acid (DHA, C22:6); arachidonic acid (AA, C20:4 ω-6)], and it is necessary to maintain cell membranes, brain functions and nerve impulse transmission under normal conditions. Therefore, *Scenedesmus* sp. is an excellent candidate for its mass production for the development/improvement of products with pharmacological action, as well as nutritional (nutraceuticals).

According to Bhalamurugan et al. [23], polyunsaturated fatty acids (DHA and EPA) are worth US$ 700 million per year, followed by β-carotene with US$ 261 million. Lutein has a market value of US$ 233 million per year and the global demand for carotenoids is expected to increase to US$ 1.8 billion by the beginning of 2020. For example, world production of fatty acids, especially EPA (omega 3), is 300 tons per year. This compound, when extracted from algae, has a higher yield than fish oil, and is cheaper [24, 25].

The protein content was estimated at 53.77% (± 0.5). Amino acids composition was also determined, with the data being presented in Table 2. Among the amino acids present, only asparagine (Asn) and glutamine (GLN) are absent, denoting the nutritional potential of algal biomass for both humans and animals, as these missing amino acids are nonessential. Commercially, *Spirulina platensis* and *Spirulina* sp. are used for the production of nutritional supplement and artificial milk formulation due to the presence of gamma-linolenic acid (GLA), besides the high protein content [22, 26]. Thus, the strain studied in this work has similar potential for the same marketing purposes.

**Table 2:** Amino acids’ composition of *Scenedesmus* sp.

Amino acid	Content (%)
Aspartate (Asp)	3.45
Glutamate (Glu)	4.44
Alanine (Ala)	3.18
Arginine (Arg)	2.87
Cysteine (Cys)	1.12
Phenylalanine* (Phe)	1.86
Glycine (Gly)	2.26
Histidine (His)	0.86
Isoleucine* (Ile)	1.25
Leucine* (Leu)	3.09
Lysine* (Lys)	2.67
Methionine* (Met)	0.66
Proline (Pro)	1.97
Serine (Ser)	1.46
Tyrosine (Tyr)	1.30
Threonine* (Thr)	1.76
Tryptophan* (Trp)	2.03
Valine* (Val)	1.82

*Essential

A strategy for the improvement and development of these microorganisms on a large scale to obtain high commercial value bioactives is associated with other microalgae applications, such as greenhouse gas fixation, effluent treatment as a pollutant bioremediation tool, biofuel production, among other processes, enabling an economically viable process in microalgae cultivation [18, 27–29].

In the pyrolysis process, the hydrocarbon molecules from biomass are cleaved into smaller molecules, in non-condensable gases like CO and CO_2_, and the solid carbon is retained in the form of coal. Generally, it is obtained 65–70% of bio-oil, which contains at least 30–40% oxygen in its composition and calorific value of approximately 20 MJ/kg [30].

**Fig. 1:**
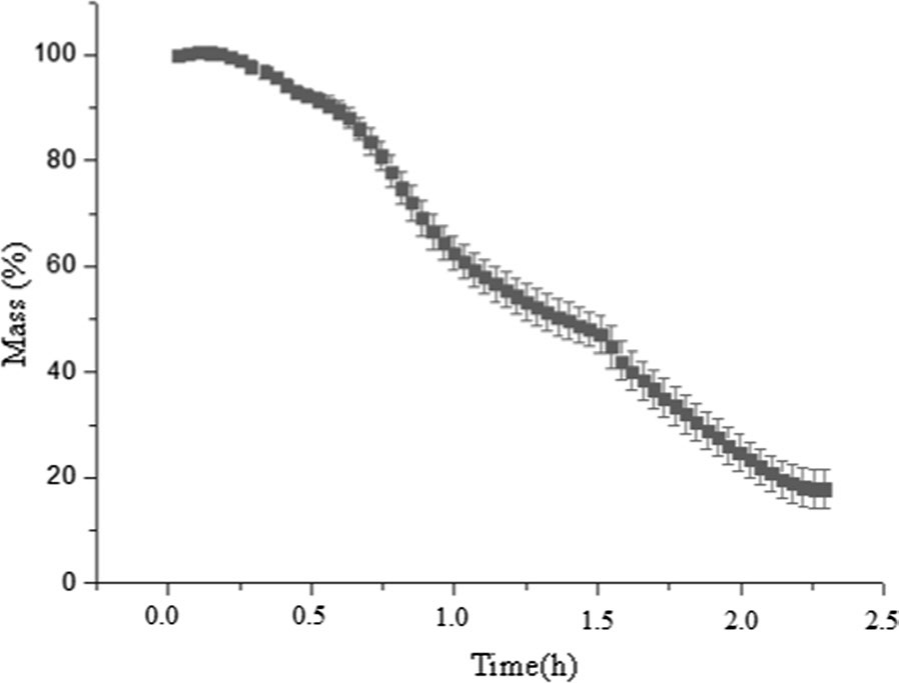
Pyrolysis of the freeze-dried algae biomass for the TGA experiments

The pyrolysis curve of *Scenedesmus* sp. biomass is presented in Fig. 1. The angular coefficients associated to the ordinate axis (corresponding to the mass variations) provide data that can be used for quantitative purposes.

The yield of liquid compounds in pyrolysis depends on the type of biomass, temperature, residence time, charcoal separation and ash content, the latter two having a catalytic effect on the decomposition of the hydrocarbons. The main issue is to optimize the temperature of the process minimizing the exposure to low temperatures that favor the formation of coal. Another possibility is to transfer heat quickly to the biomass surface [31].

In total, 82.17% (± 3.79) of the biomass underwent some thermochemical conversion process, either by pyrolysis (observed until 1.5 h in Fig. 1) or by combustion (between 1.5 and 2.25 h in Fig. 1) of the coal formed at the end of the pyrolysis. Pyrolysis was responsible for degrading 55.73% (± 3.17) of the initial biomass, while the combustion process was able to degrade 26.44% (± 2.07) of the biomass. Due to the prior pyrolysis process, it is likely that the combustion only consumed the coal resulted from the pyrolysis.

Several authors have already evaluated the pyrolysis of algal biomass. López-González et al. [32] compared the oil production of *Scenedesmus* sp. and *Nannochloropsis gaditana*. Na et al. [33] observed that different triglyceride contents in *Chlorella* sp. KR-1 could be extracted via rapid pyrolysis without the use of any solvent. Wang et al. [34] compared the direct and indirect pyrolysis of *Isochrysis*, concluding that it is possible to convert the algal biomass even after extraction of its lipids. The biomass without lipid extraction had a yield of 41.32% converted to oil, whereas the biomass that had already undergone the process of lipid extraction had a yield of 36.86% of bio-oil in the thermal decomposition of the biomass.

Pan et al. [35] studied the influence of temperature and catalysts on the slow pyrolysis of *Nannochloropsis* sp. The oil yield ranged from 19 to 31% with the presence of HZSM-5 catalysts. However, since charcoal is the main product of slow pyrolysis of biomass, the interest in using algae in combination with this process is very low today. Rizzo et al. [8] carried out the pyrolysis of *Chlorella* at 450 °C, obtaining a yield of 34% for liquids and 29% for charcoal, with the oil being less oxygenated than lignocellulosic biomass (35% vs 54%) and a higher calorific value (28.4 vs 17.2 MJ/kg).

Bridgewater [31] also suggested that no significant difference was observed in using microalgae with high lipid content in their composition for pyrolysis. This is an important result for the industrial application, because biorefineries can consider a crop without the depletion of nitrogen (the most used for accumulation of lipids), with subsequent extraction of proteins and final biomass processing thermochemically obtaining a bio-oil with different possibilities of refinement. The conversion of the liquid obtained through the processing, refining or purification to a consumable good such as electricity, heat, biofuel or chemical compounds illustrates the potential for the implementation of a thermochemical biorefinery.

**Fig. 2:**
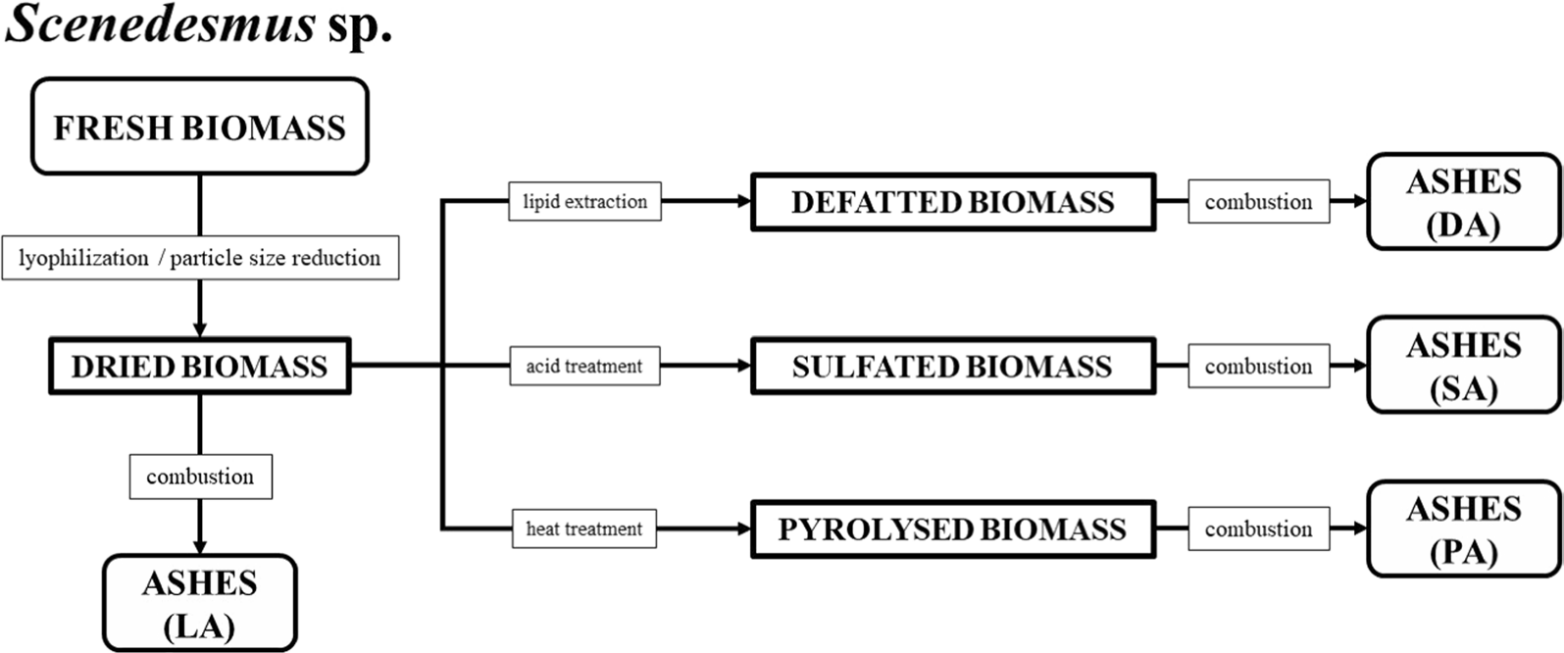
Different types of ashes samples analyzed: freeze-dried (LA), defatted (DA), sulfated (SA), pyrolysed (PA) algal biomass

After the experiments described in Fig. 2 were done, the results of ash content for *Scenedesmus* sp. are presented in Table 3.

**Table 3:** Ash contents present in the biomass of *Scenedesmus* sp.

*Scenedesmus* sp.	Content (%)	Standard
Freeze-dried biomass (LA)	14.32 (± 0.14)^a^	ASTM E1755-01
Defatted biomass (DA)	14.47 (± 0.30)^a^	ASTM E1755-01
Pyrolyzed biomass (PA)	16.81 (± 2.47)^a, b^	ASTM D7582-15
Sulfated biomass (SA)	17.81 (± 0.15)^b^	ASTM D3516-89 and Brazilian Pharmacopeia (5.2.10)

Different letters indicate significant difference (*p* < 0.05) by Tukey test

Knowing the content of ash and its elemental composition is essential in the development and improvement of equipment and thermochemical conversion methodologies. The method for the determination of sulfated ash is due that volatile compounds (organic and inorganic) are released when the biomass is incinerated, while non-volatile inorganic compounds remain in the ashes. This difference emitted in thermochemical processes is referred to as volatile ash and the remaining material is the bottom ash.

Unlike the incineration, the volatilization of alkali metal halides (NaCl, KCl, etc.) is avoided by the addition of concentrated sulfuric acid, transforming these substances into alkaline sulfides that have low volatility [36], resulting in the higher value for the sulfated biomass presented in Table 3.

It is observed that the value of sulfated ash was 18.5% higher than ash values without the process of biomass sulfation (freeze-dried biomass). This difference observed should be considered, depending on the thermochemical conversion path and the elemental composition of the ashes. The value of ash content of pyrolysed biomass can be explained as an incomplete combustion of the coal resulted in the pyrolysis process.

During combustion, a fraction of the ash-forming compounds is volatilized and released into the gas phase. This volatilized fraction is dependent on the characteristics of the fuel, the gas atmosphere and the combustion technology in use [37]. Excluded minerals will undergo transformation or decomposition, melting and solidification during combustion, while minerals or organically bound elements will undergo decomposition, fragmentation, vaporization and condensation [38]. The main constituents of any biomass are C, O and H. N and S are present in smaller amounts. The main elements of the biomass ashes are K, Ca, Cl, Si and P, Mg, Na, and Ti [37, 39, 40].

**Fig. 3:**
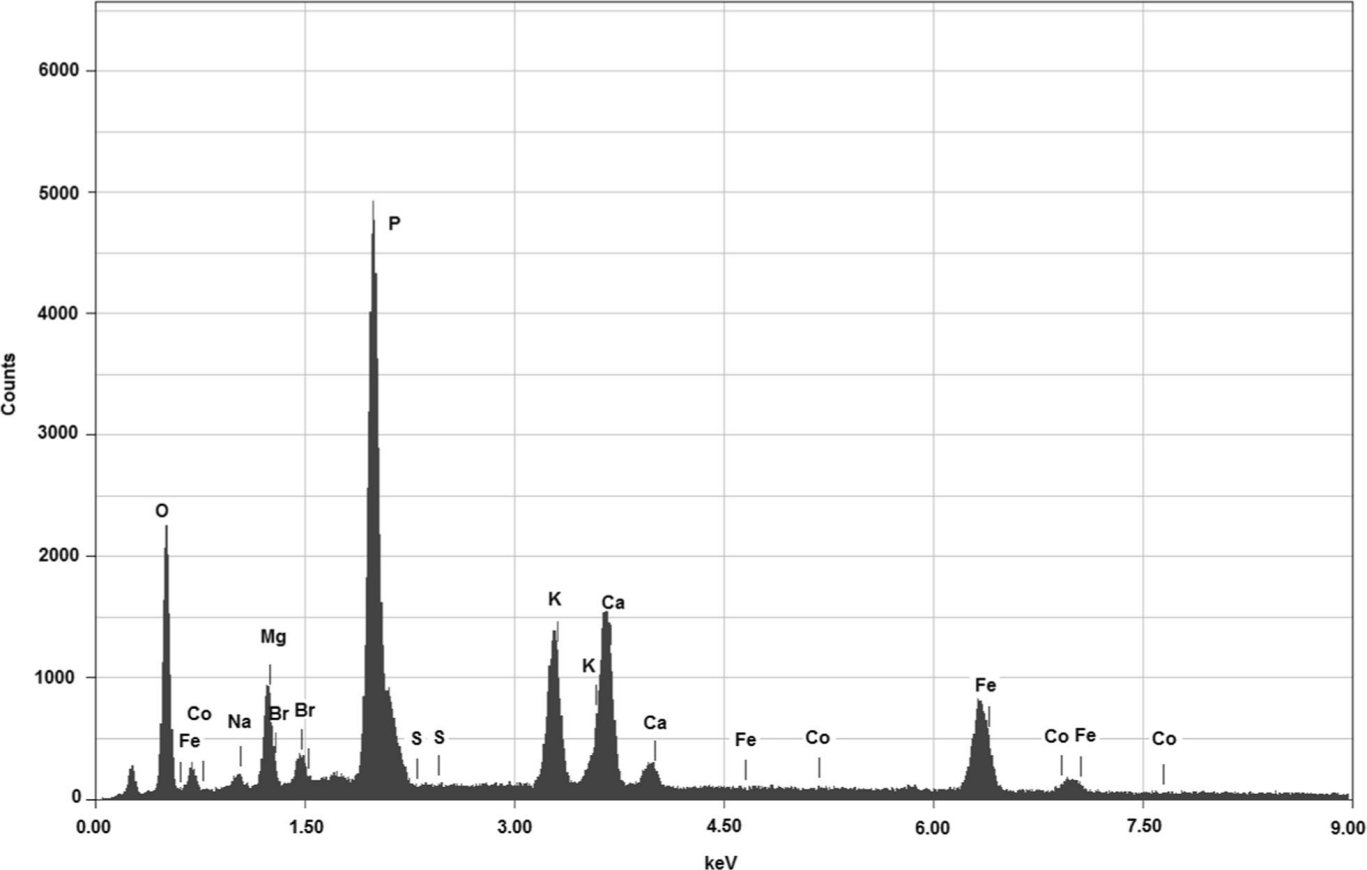
Data obtained by SEM–EDS experiment with *Scenedesmus* sp. freeze-dried biomass

The data of ash element content (mass percentage, Fig. 3) determined from samples of the pyrolysed, defatted, freeze-dried and sulfated biomass are presented in Table 4. Element concentrations near detection level of lower limit of the equipment could not be quantified (< 1%: Na, Si, Cr, Mn, Ni, Cu, Zn, Se and V). Others elements (C, Au, Al) were used in the procedure, being disconfirmed their content.

**Table 4:** Element content in the biomass ashes of *Scenedesmus* sp.

Elements (% by mass)	Freeze-dried		Defatted		Pyrolyzed		Sulfated	
	Mean	SD	Mean	SD	Mean	SD	Mean	SD
O	40.25^a^	0.27	39.32^a^	0.48	40.30^a^	0.33	41.56^a^	4.04
Mg	6.16^a^	0.34	8.46^b^	0.35	7.10^a,c^	0.25	8.46^b,c^	1.76
P	20.84^a^	0.33	19.27^b^	0.45	20.74^a^	0.46	8.58^c^	1.50
S	ni		ni		ni		11.51	1.76
K	7.61^a^	0.19	7.10^b^	0.19	7.55^a,b^	0.20	2.73^c^	0.65
Ca	8.65^a^	0.26	7.73^b^	0.18	7.91^b^	0.29	3.12^c^	0.60
Fe	11.01^a^	0.71	11.32^a^	0.25	10.50^a^	0.67	15.43^b^	3.45
Co	1.69^a^	0.31	1.94^a^	0.46	1.53^a^	0.30	2.51^a^	1.57
Br	2.14^a^	0.09	3.21^a^	0.55	2.34^a^	0.47	4.69^a^	3.95

Different letters indicate significant difference (*p* < 0.05) by Tukey test

*ni* not identified

According to Mobin et al. [41], microalgae are known to be high in mineral content. More than 6.7% of the dry weight of microalgae *Spirulina* is ash. The strain of this work presented a higher value than 14.32% (Table 3). A number of conditioners influence its mineral composition, such as cultivation temperature, salinity, physiological state of the strain, geographic distribution, among others.

Galvão et al. [42] presented a work with an up-to-date overview of the available analytical techniques applied to particulate matter (PM) characterization, which could also be applied to the characterization of sediments and soils. According to them, among X-ray-based techniques for PM characterization, Energy Dispersive X-ray is one of the most applied and well-established methods that could be used for the semi-quantitative elemental composition, because the radiation emitted by ionized atoms emitted contains a selective, qualitative and quantitative information of the elemental constituents present in the sample. The authors [42] also highlight that EDS is essentially a non-destructive technique, although some lighter elements and semi-volatile compounds can be lost due to X-ray radiation and under vacuum analysis. The minimum requirement is that the sample should be homogeneous. The EDS technique is used with different objectives, as studying human health impacts and influence on visibility, and presents an easy operation and relatively low-cost X-ray-based technique, being non-destructive and requiring minimal sample handling [42]. This reasoning allows us to say that the same technique can be used in the elemental characterization of ashes present in microalgae biomass after thermochemical processes.

Among the samples evaluated, after subtracting O value, P is the element with the highest percentage for the fractions evaluated, except for sulfated ash, in which the most abundant element was Fe (Table 4). Overall, freeze-dried, defatted and pyrolyzed ashes had a similar element content, with the only exception being a lower content of P in the defatted biomass ashes. The sulfated sample presented a unique composition, with the presence of S, higher percentage of Fe, and lower value for P, compared to other treatments. The Fe in the microalgae is acid-soluble, possibly in the form of FeS, what could explain the higher content [10]. The high content of S in algae contributes for greater SO_X_ emissions, generation of fine particulates and less stable and mobile sulfates; and deposit formation, agglomeration, slagging and corrosion, although part of this value may be residues from the treatment with sulfuric acid [43].

Kutchko and Kim [44] studied the fly ash samples of a pulverized coal fired boiler and observed a number of iron-rich spheres. The majority of the iron-rich spheres consisted of an iron oxide phase mixed with an amorphous alumino-silicate phase. It can result in the observed sulfated ashes’ element content.

The vaporization of P, one of the most abundant elements in the biomass, and subsequent chemical reactions are responsible for a great part of the dirtiness, sulfation, corrosion and formation of silicates in biomass boilers. Therefore, their presence may imply a shorter life expectancy [41]. Phosphate, in the freeze-dried, defatted and pyrolyzed microalgae is mainly water soluble, and this can be the reason for the lower value observed in the defatted biomass [10].

Complex fuels contain high levels of K and other alkalis and alkaline metals that vaporize and react with other elements as they pass through the boiler, partially condensing to form deposits on metal and refractory surfaces [39]. In addition, according to Miles et al. [39], knowing the K content in the biomass is important since it can indicate the possible ash melting or its deposition through vaporization processes and condensation. K reacts during combustion and mixes with other elements such as chlorine, sulfur and silicon. It has been demonstrated by Gómez et al. [45] that K and P content of ashes, together with temperature, interacts in the pyrolysis reaction and affects the chemical composition and physical characteristics of bio-oil.

During the thermochemical decomposition of the biomass, corrosion may occur inside the boilers, mainly due to the presence of Cl. Chlorine reacts with the alkaline material to form relatively volatile and stable alkaline chlorides. Condensation of the chlorides on relatively cold surfaces in the presence of S often leads to the formation of sulfates. Cl also causes corrosion at low temperatures through the formation of acid gases [39].

There are two types of oxides in ash composition: the basic ones, such as Na_2_O, K_2_O, MgO, CaO and Fe_2_O_3_; and acids, such as SiO_2_, Al_2_O_3_ and TiO_2_. The relationship among these types of oxides is the simplest index to predict the tendency of slag formation and sintering. The basic compounds of the ash reduce the melting temperatures, whereas the acid oxides provide their increase [40]. Table 5 shows the oxides formed from the elements present in the evaluated samples.

**Table 5:** Oxide content in the biomass ashes of *Scenedesmus* sp.

Elements (% by mass)	Freeze-dried		Defatted		Pyrolyzed		Sulfated	
	Mean	SD	Mean	SD	Mean	SD	Mean	SD
MgO	10.21^a^	0.57	14.03^b^	0.58	11.77^a^	0.42	14.03^b^	2.91
P_2_O_5_	47.76^a^	0.73	44.14^b^	0.99	47.52^a^	1.01	19.65^c^	3.26
SO_3_	ni		ni		ni		28.75	4.40
K_2_O	9.16^a^	0.22	8.55^b^	0.22	9.10^a^	0.23	3.29^c^	0.75
CaO	12.10^a^	0.35	10.82^b^	0.24	11.07^b^	0.39	4.37^c^	0.80
Fe_2_O_3_	14.16^a^	0.91	14.56^a,b^	0.33	13.51^a,b^	0.86	19.96^c^	4.40
CoO	2.14^a^	0.39	2.46^a^	0.58	1.95^a^	0.38	3.19^a^	2.00
Br	2.14^a^	0.09	3.21^a^	0.55	2.33^a^	0.47	4.69^a^	3.95

Different letters indicate significant difference (*p* < 0.05) by Tukey test

*ni* not identified

The higher content of alkaline earth elements (Ca and Mg) in algae can indicate the use in soil amendment and fertilization; production of construction materials, adsorbents and ceramics; synthesis of minerals and recovery of valuable components. In addition, these fuels could produce suitable bed materials that may reduce the need to use additives in fluidized bed combustion chambers [43].

Alkaline and halogen elements (Br, K, Na and Cl) represent a challenge in the thermochemical conversion of algae, due to volatilization and formation of many dangerous enhanced fine-particulate emissions; greater quantity of water-soluble fraction; generation of low-melting eutectic phases and low ash-fusion temperatures; increased deposit formation, fouling, agglomeration, slagging and corrosion; and among others problems. Minor oxides such as CaO, MgO, Na_2_O and K_2_O also indicate a significant influence on the fusibility behavior of the ashes, because the increase of these oxides contributes in the formation of phases with high fluidity [43, 46].

According with the literature [43], the main ash-forming oxides in algae ash (macro, green, brown, red, marine, in percentage) are SiO_2_ (20.38), SO_3_ (18.81), Cl_2_O (16.13), K_2_O (15.71), Na_2_O (13.56), CaO (7.28), MgO (3.42), P_2_O_5_ (1.80), Fe_2_O_3_ (1.55), Al_2_O_3_ (1.23) and TiO_2_ (0.13). These same authors stated elements such as Au, B, Br, Cl, I, K, Mg, Na, P, and Sr show the highest enrichment factors (above 1 order of magnitude) in the algae in relation to coal. However, these authors noted a low concentration of Fe in algae, which was not observed in this work. The presence of Fe and S can indicate an increase of the ash-fusion temperatures, which could decrease the slag formation. High iron content may indicate the use of *Scenedesmus* sp. for cultivation in waste water, due to its potential use of algae for heavy metal bioremediation, as the ash samples obtained presented a significant value of this element in the studied samples [47, 48].

Chen et al. [47] showed that algal biomass with a minor ash content could effectively improve the biocrude oil quality in terms of higher heating values (from 25.8 MJ/kg to 29.2–32.3 MJ/kg) with lower boiling points. Although, the ash could provide denitrogenation and catalyze the formation of hydrocarbons under hydrothermal liquefaction processes.

Coutinho and Vieira [49] indicated that the incorporation of biomass ash into red ceramics is promising and could become an environmentally correct destination for this residue. Asquer et al. [50] observed that biomass ash could be used as an additive in the composting process of organic fraction of municipal solid waste, because it favors the aerobic degradation by acting as a physical conditioner. Zhou and Ma [51] suggested that ash biomass promotes sintering behavior in coal. All these studies indicate possible destinations for biomass ashes obtained in thermochemical processes.

## Conclusions

In the present study, experimental results were obtained, from which some of the following conclusions could be drawn, divided by the following points: lipid content and pyrolysis biomass of *Scenedesmus* sp; ash content from freeze-dried, defatted, sulfated and pyrolysed biomass of *Scenedesmus* sp.; identification of elements and oxides in ash samples.

The information present in this work allows to conclude that the sonication-assisted lipid extraction methodology is a valid methodology for obtaining algae lipids. *Scenedesmus* sp. presented 16.72% content. In addition, its fatty acid composition indicates a high content of polyunsaturated acids (67.93%), especially GLA (52.98%) and LA (13.46%), which may be used in the formulation of products with high value-added (nutraceuticals and cosmeceuticals). Therefore, it is suggested the extraction/purification of these compounds and then the production of biodiesel from the residual oil. Among the amino acids present, only asparagine and glutamine (nonessential) are absent, denoting the nutritional potential of algal biomass for both humans and animals.

The majority of the biomass underwent some thermochemical conversion process, either pyrolysis or combustion of the coal formed at the end of the pyrolysis. The use of microalgae as a feedstock for pyrolysis has many advantages over other renewable and conventional sources of energy, such as the non-use of arable land and the stabilization of a chain of co-products that can be obtained. Compared to other conversion technologies, the use of algal biomass pyrolysis is well studied and has presented reliable and promising results that could lead to commercial exploitation if the concept of thermochemical biorefinery is applied.

The content of the sulfated ash of 17.81 ± 0.15% is of extreme importance, because especially given little information available in the literature. Depending on the thermochemical conversion route and the elemental composition of the ashes, the vaporization and subsequent chemical reactions are responsible for much of the soil, sulfation, corrosion and formation of silicates in biomass boilers, which can cause a decrease in process efficiency.

The SEM-EDS technique is useful for the identification of mineral compounds in ashes. The main limitations relate the low sensitivity. The DRX analyzes allowed to quantify Mg, P, S, K, Ca, Fe, Co and Br, as well as oxides like MgO, P_2_O_5_, SO_3_, K_2_O, CaO, Fe_2_O_3_ and CoO in the set of ashes analyzed. These data indicate possibility to determine and make feasible a thermochemical conversion route to obtain fuel products with the algal biomass.

## Methods

### Microalgal biomass

The freshwater microalgae selected for this study was *Scenedesmus* sp. (Chlorophyceae), whose fresh biomass was kindly donated by the company *Algae Biotecnologia*^*®*^. The biomass was received in January 2018 and kept frozen at − 22 °C for the proposed analysis. Based on information provided by the company, the production of algal biomass (autotrophic cultivation) was carried out in batch, in a vertical thin-layer tubular reactor, under air bubbling, with natural light and average temperature of 29 °C. The culture medium used was M8-a in fresh water, with a final volume of 250 L [52]. The cultivation was carried out in Piracicaba—SP, Brazil during 04 days.

### Ashes’ obtainment from different treatments

To obtain the different ash types, the biomass previously underwent a process of lyophilization (temperature − 81 °C and vacuum of 0.024 mBar). The frozen biomass was carried out to determine the dry weight, calculated by gravimetric analysis [53]. Then, the particle size reduction/standardization process was carried out in analytical mill with a 0.5-mm (35 mesh) separation screen. The freeze-dried samples were frozen immediately and stored in a freezer (− 22 °C) for further analysis. Before starting the analysis procedures, the samples were allowed to reach room temperature. After the standardization of samples (Fig. 2), the procedures were performed as follows.

### Ashes from freeze-dried biomass (LA)

The ash content in lyophilized and defatted biomass was quantified following the ASTM E1755-01 standard [54]. Initially, 50-mL porcelain crucibles were calcined in a furnace at 575 (± 25) °C for a period of 3 h. Then, they were transferred to a desiccator until reach room temperature, before weighing in analytical balance for the determination of the empty crucible mass. Subsequently, approximately 1.0 g of the lyophilized biomass of *Scenedesmus* sp. was added to the crucibles. They were placed in a muffle oven at room temperature and heated to 250 °C for 30 min. This step is necessary to avoid the sample being ejected or having a more intense combustion due to the presence of volatiles, causing loss of biological material. After the 30-min period, the samples were heated to 575 (± 25) °C, for 1 h. After the process, the crucibles were placed in a desiccator, to reach room temperature before weighing. This procedure of heating and subsequent cooling in the desiccator was repeated until the final mass of the crucible containing the ashes of the biomass did not vary more than 0.5 mg. The experiments were performed three times.

The fatty acids’ and amino acids’ composition of *Scenedesmus* sp. was determined by the company, *bioMérieux Brasil*^®^. Approximately 10 g of lyophilized biomass was sent to their laboratory, which determined the fatty acid profile through gas chromatography–mass spectrometry and the amino acid profile through high-performance liquid chromatography.

### Ashes from defatted biomass (DA) and sonication-assisted lipid extraction

The lipid extraction methodology used was developed by the group in a previous work [7]. The freeze-dried biomass (100 mg) was transferred to a 50-mL centrifugation tube, to which 20 mL of *n*-hexane (analytical grade) was added. The samples were mixed on a vortex and submitted to an ultrasonic ice/water bath during 20 min. After sonication, the samples were centrifuged at 4500 rpm for 10 min and the supernatant was transferred with the aid of a pipette to a 50-mL round-bottomed flask of known mass; the solvent was removed through rotary-evaporation. Cell debris was resubmitted to *n*-hexane extraction. This procedure was repeated until completion of lipids extraction (6 times). After concentrating the extracted oil, the round-bottomed flask was lyophilized to achieve the complete removal of solvent and moisture on the sample. Gravimetric analysis was used to determine the lipid content. The defatted biomass was once again frozen and freeze-dried. This material was later used to obtain the defatted biomass ashes, using the same methodology for LA.

### Sulfated ashes (SA)

For the sulfated ashes, the Brazilian Pharmacopeia (standard 5.2.10, 2010) [55] and ASTM D3516-89 standard [56], which provides different methods for the determination of ashes, were used for the determination of sulfated ash per residue by incineration. Sulfated ash comprises the non-volatile residue on incineration in the presence of sulfuric acid. This test intended to determine the content of constituents or inorganic impurities in organic substances. As in traditional ash analysis, the crucibles were calcined in a furnace at 575 (± 25) °C for 3 h, placed in a desiccator and weighed in analytical balance after reached room temperature. About 1.0 g of lyophilized algae biomass was added into the crucibles. Then, 1 mL of concentrated sulfuric acid (analytical grade) was added to the samples and the crucibles were heated to 575 (± 25) °C in the furnace for 1 h. After cooling in desiccator, the crucibles were weighed again. This procedure was repeated by changing the heating time to 30 min and without the addition of sulfuric acid. After the period, the crucibles were cooled and their masses checked. This procedure was repeated until the final mass of the crucible containing the sulfated ash did not vary more than 0.5 mg. The experiments were performed in three replicas.

### Thermogravimetric analysis—pyrolysis (PA)

To determine the effects of temperature and heating rate during pyrolysis, the freeze-dried samples were pyrolysed through a thermogravimetric analysis (TGA). The experiments were realized in a TGA Macro analyzer (*Navas Instruments* TGA-2000 Series). For this experiment, the proposed methodology was based on the ASTM D7582-15 standard [57]. Initially, 1 g of lyophilized biomass was added in previously weighed crucibles. The experimental procedure performed to evaluate the pyrolytic behavior of the algal biomass was heating rate of 20 °C/min; gas flow (N_2_) of 10 L/min and 650 °C as final temperature. After achieving this temperature, the gas was changed to O_2_, with 4 L/mL, until no mass variation was observed, to obtain the ashes from the pyrolysed biomass.

### Analysis of elemental ash composition

The methodology to determine the ash element content from samples of the pyrolysed, sulfated, defatted and freeze-dried biomass was developed by Tiburcio et al. [58]. Aluminum stubs were demarcated in three sections for the analyses. Then, the ash samples were deposited, with the assistance of a spatula, over double-coated carbon tapes (~ 7 × 8 mm), previously fixed to the marked sections The prepared stubs were stored in a glass desiccator for further sputter coating with gold (15 nm in diffuse mode; *Leica EM ACE200*).

The analyses were performed using the SEM–EDS (scanning electron microscope fitted with energy dispersive spectroscopy) technique (*JCM*-*6000*-*OPT Neoscope II EDS Analysis Option*). The operating conditions of the equipment were high vacuum, secondary electron detector; 15 kV voltage; high filament intensity; and high current density. Spectra were obtained from an image standardization with magnification of 200 times. For each stub, three points were randomly selected by section.

### Statistical analysis

Data are expressed as means, with their standard deviation. The results were compared by analysis of variance (ANOVA), using the Tukey test, considering *p* < 0.05 as a statistically significant difference, using the *OriginPro 8.1* software.

## Data Availability

The datasets used and/or analyzed in this study are available from the corresponding author upon reasonable request.

## Abbreviations

LA: Freeze-dried biomass
DA: Defatted biomass
PA: Pyrolyzed biomass
SA: Sulfated biomass
PM: Particulate matter
SEM–EDS: Scanning electron microscope fitted with energy dispersive spectroscopy
TGA: Thermogravimetric analysis
